# Supplementary material for: Transport of bound quasiparticle states in a two-dimensional boundary superfluid
Source: Nat Commun. 2023 Nov 2;14:6819. doi: 10.1038/s41467-023-42520-y (PMC10622538; doi:10.1038/s41467-023-42520-y)
Supplement: Supplementary file 1 — Supplementary Information [file 41467_2023_42520_MOESM1_ESM.pdf]

Supplementary information for transport of bound quasiparticle states in a two-dimensional boundary superfluid

S. Autti[1],* R. P. Haley[1], A. Jennings[1],† G. R. Pickett[1], M. Poole[1], R. Schanen[1],
A. A. Soldatov[2], V. Tsepelin[1], J. Vonka[1],‡ V. V. Zavjalov[1], and D. E. Zmeev[1]
[1]*Department of Physics, Lancaster University, Lancaster LA1 4YB, UK. and*
[2]*P.L. Kapitza Institute for Physical Problems of RAS, 119334 Moscow, Russia*

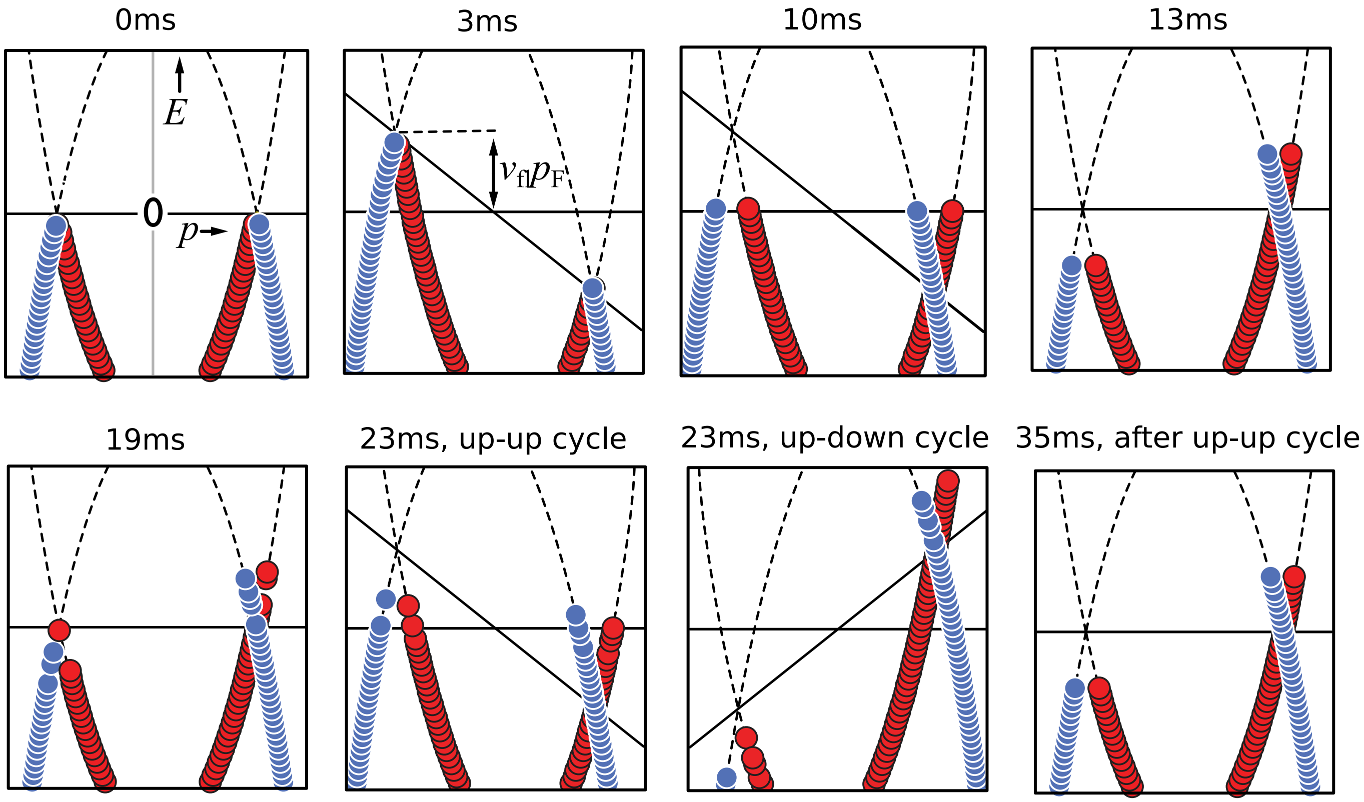

FIG. S1. **Bound quasiparticle dynamics:** The crossbar up-up cycle shown in Fig. 3 starts at $t = 0$ ms with an equilibrium bound state population (red and blue disks illustrate quasiparticles). At zero temperature, no quasiparticles are above the Fermi energy (here at $E = 0$). After the acceleration ($t = 3$ ms), the superflow v_{fl} along the surface has shifted the states by $\pm p_F v_{fl}$. The quasiparticles energetic enough to escape to bulk scatter away, and diffusion equilibrates the rest of the spectrum. Here we have assumed this process is finished at $t = 10$ ms. The wire is then brought to a halt ($t = 13$ ms). The diffusive redistribution of the quasiparticles is determined by the time constant τ ($t = 19$ ms). Moving the wire soon thereafter in either the same direction or the opposite direction ($t = 23$ ms) and recording the resulting quasiparticle emission reveals the population dynamics. At $t = 35$ ms the wire is stationary again. Note that the spectral evolution is shown here for a Dirac spectrum at zero temperature, the effect of the bulk escape is neglected for simplicity, and the direction of momentum is assumed to be uniformly along the surface. These choices allow for compact visualisation, but they should not be used for detailed calculations.

---

* s.autti@lancaster.ac.uk
† Current address: RIKEN Center for Quantum Computing, RIKEN, Wako, 351-0198, Japan
‡ Current address: Paul Scherrer Institute, Forschungsstrasse 111, 5232 Villigen PSI, Switzerland
